# A pilot study for augmenting atomoxetine with methylphenidate: safety of concomitant therapy in children with attention-deficit/hyperactivity disorder

**DOI:** 10.1186/1753-2000-1-10

**Published:** 2007-09-27

**Authors:** Gabrielle A Carlson, David Dunn, Douglas Kelsey, Dustin Ruff, Susan Ball, Lisa Ahrbecker, Albert J Allen

**Affiliations:** 1 Department of Child and Adolescent Psychiatry, Stony Brook School of Medicine, Stony Brook, New York 11794-8790, USA; 2 Department of Psychiatry, Indiana University School of Medicine, Indianapolis, Indiana 46202, USA; 3 Lilly Research Laboratories, Eli Lilly and Company, Indianapolis, Indiana 46268, USA

## Abstract

**Background:**

This study examined augmenting atomoxetine with extended-release methylphenidate in children whose attention-deficit/hyperactivity disorder (ADHD) previously failed to respond adequately to stimulant medication.

**Methods:**

Children with ADHD and prior stimulant treatment (*N *= 25) received atomoxetine (1.2 mg/kg/day) plus placebo. After 4 weeks, patients who were responders (*n *= 4) were continued on atomoxetine/placebo while remaining patients were randomly assigned to either methylphenidate (ATX/MPH) (1.1 mg/kg/day) or placebo augmentation (ATX/PB) for another 6 weeks. Patients and sites were blind to timing of active augmentation. Safety measures included vital signs, weight, and adverse events. Efficacy was assessed by ADHD rating scales.

**Results:**

Categorical increases in vital signs occurred for 5 patients (3 patients in ATX/MPH, 2 patients in ATX/PBO). Sixteen percent discontinued the study due to AE, but no difference between augmentation groups. Atomoxetine treatment was efficacious on outcome measures (*P *≤ .001), but methylphenidate did not enhance response.

**Conclusion:**

Methylphenidate appears to be safely combined with atomoxetine, but conclusions limited by small sample. With atomoxetine treatment, 43% of patients achieved normalization on ADHD ratings.

## Introduction

Pharmacological interventions are considered first-line treatment for attention-deficit/hyperactivity disorder (ADHD) [1]. The U.S. Food and Drug Administration (FDA) has approved stimulant medications and atomoxetine for the treatment of ADHD. Stimulant medications have a long-established use and may partly work through the dopaminergic neurotransmitter system. Atomoxetine is a relatively new, nonstimulant medication that is a potent selective inhibitor of the presynaptic norepinephrine transporter. Dysregulation in dopaminergic and noradrenergic systems has been implicated in the pathogenesis of ADHD [2].

Although stimulants and atomoxetine each have been established as an effective monotherapy for the treatment of ADHD, a number of clinical situations may arise that would suggest the strategy of augmenting or combining the 2 treatments. Augmentation often occurs when a patient responds to an initial intervention but continues to have residual symptoms that cause significant distress or impairment. Tolerability concerns also play a role in the decision to add a second medication. After reaching a specific dose level, a patient may begin to experience adverse events if there is further increase in dosage. For example, a child could need additional dosing of a stimulant medication for management of symptoms in the evening, but such an increase might lead to insomnia. Thus, the different profiles of the medications can be used together to manage adverse events within the goal of improving the psychiatric illness.

Reflecting these real-world situations, rates of concomitant psychotropic medications have increased substantially over the past decade. Among youths being treated with stimulants, Bhatara et al [3] reported that prescription patterns of combining stimulants with other psychotropics have increased from 4.8% in 1993–94 to 24.7% in 1997–1998. Despite the increase in the practice, empirical support for the safety and efficacy of concomitant treatments remain limited [4]. Controlled trials for combination treatment of ADHD have been conducted primarily to combine stimulants with tricyclic antidepressants. An earlier study of desipramine and methylphenidate (MPH) suggested synergistic effects for the combination that were superior to either intervention alone [5]. However, the combination treatment also was associated with more adverse events than the monotherapies [6]. Case reports of combination atomoxetine and stimulant treatments have provided promising findings, but these reports also underscore the need for systematic study [7].

Approximately 30% of children fail to respond adequately to a trial of stimulant medication [8]. As children with an inadequate response to stimulants are likely to be prescribed atomoxetine, the present study examined the initial response to atomoxetine followed by augmentation with either extended-release (OROS) methylphenidate (MPH) or placebo. Our primary objective was to examine the safety of this augmentation strategy for atomoxetine in children with ADHD.

## Methods

### Patient selection

Patients were children ages 6 through 12 recruited from 5 outpatient centers. To be eligible for the study, children had to meet the following criteria: a DSM-IV diagnosis of ADHD, any type; a rating on the ADHD Rating Scale Version IV Parent Reported-Investigator Rated version (ADHDRS-IV-Parent:Inv) [9] of at least 1.5 standard deviations above age and gender norms; and a severity rating of at least moderate on the Clinical Global Impressions Severity Scale (CGI-S). Additionally, they must have experienced a prior history (preceding 12 months) of insufficient response to an adequate stimulant trial, which was defined as a gradual titration of stimulant medication for at least two weeks at specified doses for each of the medication. Inadequate response was determined by the child's prescribing physician who also documented his or her opinion that a change in treatment was needed.

Children were excluded from participating in the study if they weighed less than 22 kg or more than 60 kg at study entry; had any other Axis I diagnosis, including pervasive developmental disorder, mood, or anxiety disorder; had any medical conditions that would contraindicate the use of either atomoxetine or extended-release methylphenidate, or used any concomitant psychotropic or excluded medications. The presence of comorbid oppositional defiant disorder was not an exclusion criterion. Children who had a history of intolerance or nonresponse to atomoxetine were excluded because of the ethical reason that they should not be enrolled in an augmentation study in which they have demonstrated inadequate response to both treatments. All patients were required to be free of any excluded medications for at least 5 days prior to baseline ratings and randomization.

Each site's institutional review board approved the conduct of the study, which was developed in accordance with the ethical standards of Good Clinical Practice (GCP) and the Declaration of Helsinki, as revised in 2000 [10]. Parents or legal guardians of all subjects provided written informed consent, and the patients gave verbal assent to participate in the study.

### Assessment procedures

At the initial screening visit, patients underwent a thorough diagnostic and medical examination. The diagnosis of ADHD was determined using the semi-structured clinical interview, KIDDIE-SADS-PL version [11], and was confirmed by a child psychiatrist. The medical evaluation included medical history, physical exam, routine chemistry, hematology, urinalysis, and electrocardiograms.

During the course of treatment, safety was assessed by obtaining vital signs, weight, spontaneous adverse event reports, and concomitant medications at each visit. Illness severity measures included the ADHDRS-IV-Parent:Inv; Clinical Global Impression Scale – Improvement (CGI-I) [12] ratings; the Weekly Parent Ratings of Evening and Morning Behavior-Revised (WREMB-R); and the Conners Parent Rating Scale Revised, Short-Form (CPRS-R:S) [13]. With the exception of the WREMB-R, these outcome measures have been established as valid and reliable within the ADHD field, and they are scored so that higher scores indicate greater symptom severity. The WREMB-R is a more recent instrument in which a parent rates 11 behaviors for their severity during the morning and evening hours, and it has shown sensitivity to treatment effects in prior clinical trials [14]. Overall improvement also was rated by clinicians using the CGI-I, which consists of a 7-point scale where 1 = "very much improved" and 7 = "very much worse". The safety and efficacy measures were repeated again at the end of treatment or when patients discontinued the study.

### Study design

The study design consisted of 3 sequential phases: an evaluation/screening phase, a double-blind 4-week acute treatment (study phase 1); and a 6-week, double-blind, combination treatment phase (study phase 2). At visit 2 (treatment week 1), all patients were started on open-label atomoxetine and given a pill placebo. Atomoxetine was titrated to a target dose of 1.2 mg/kg/day (maximum dose 1.4 mg/kg/day). Neither the investigator nor the patients knew when the onset of active augmentation would occur as the investigator's protocol did not specify the timing of active augmentation. Another protocol that specified the onset of augmentation was mailed directly to the investigator's institutional review board for full disclosure. After 4 weeks on placebo, patients' illness severity was compared with their baseline using the above scales. If patients were rated on the CGI-I scale as 1 or 2 (much or very much improved), they were classified as remitters and were maintained on placebo. If patients continued to have substantial symptoms, they were then randomly assigned via an interactive voice response system to receive either extended-release methylphenidate or placebo. Dose for OROS methylphenidate was titrated to a target dose of 1.08 mg/kg/day (maximum dose 1.2 mg/kg/day). All patients continued with their open label atomoxetine dose during the active augmentation treatment phase. During study phase 1, patients were seen after 14 days and then weekly for 2 visits (4 weeks total). During study phase 2, they were seen weekly for 2 weeks, and then 1 month later (6 weeks).

### Statistical methods

The power calculations for the study were based on the primary objective of safety by estimating the number of patients who would be required to demonstrate a safety signal in categorical changes in vitals. Based on the assumption of 5% of patients would have a categorical change in vitals on atomoxetine, 85 subjects were estimated to be required for an 80% power to detect a eight-fold increase in categorical changes. However, it was difficult to find a sample of children who had stopped stimulant treatment, were not intolerant of stimulants, and had not already been treated with atomoxetine. Thus, the final sample size was 25, which caused the study to be underpowered to detect categorical differences between groups.

Safety and tolerability outcomes were reported with frequency counts. Paired *t *tests were used to examine whether mean changes from baseline to the end of study phase 1 and mean changes from baseline to study phase 2 were significantly different from zero. Categorical changes in vital signs were defined as follows: (1) for diastolic and systolic blood pressure, an increase of at least 5 mmHg to above the 95^th ^percentile based on age, gender, and height-adjusted National Institute of Health norms [15]; (2) for pulse, an increase of at least 25 to a value of at least 110 bpm.

Efficacy outcome measures were conducted on the intent-to-treat sample using a last-observation-carried-forward method. Patients were classified into 3 groups: atomoxetine/methylphenidate, atomoxetine/placebo excluding early responders (who were not randomized), and atomoxetine/placebo including early responders. Efficacy was analyzed using a repeated measure analysis of covariance (ANCOVA) comparing changes on the ADHDRS-IV-Parent:Inv total score from initial baseline, at the end of study phase 1, and the end of study phase2. The initial baseline score was the covariate, and treatment and investigator were fixed effects. Effect sizes were also calculated on the secondary outcome measures to determine the overall treatment response (10-week treatment from baseline to study end-point) and the incremental effect size (6-week double-blind randomization to study end-point).

Efficacy was examined descriptively by classifying patients individually based on their *T*-score obtained from the ADHDRS-IV-Parent:Inv total at the end of study phase 1 and at the end of study phase 2. Patients were classified as normalizers if their scores at the end of the study phases were within 1 standard deviation of the normal range (i.e., *T*-score ≤ 60). The frequency of normalization was then summed to determine the rate of those who did not improve, who transiently improved (normalized at end of study phase 1, but not study phase 2), or obtained/maintained improvement (normalized at end of both study phases).

## Results

Twenty-five children met the inclusion criteria. All but 1 patient (who withdrew) entered into the 4-week treatment phase with atomoxetine and placebo (PBO). Mean age of this sample was 9.6 years old (sd = 1.8); 83% were male, and 83% were Caucasian. Nineteen (79%) met the criteria for ADHD combined type, and 12 (50%) had a comorbid oppositional defiant disorder. Of the 25 children, 4 children discontinued the study prior to randomization (includes the child who withdrew); 4 were classified as early responders during the initial atomoxetine 4-week treatment and were not randomized (RESP-ATX/PBO); 9 children were randomly assigned to extended-release (OROS) methylphenidate (ATX/MPH); and 8 were randomly assigned to placebo (ATX/PBO) augmentation. Reasons for early discontinuation was for adverse events (n = 2), perceived lack of efficacy (n = 1), and physician decision (n = 1).

### Safety and tolerability

Fourteen treatment-emergent adverse events (TEAEs) occurred during the study (Table 1). Overall, there were numerically fewer TEAE in the ATX/MPH group compared with the ATX/PBO group. One TEAE was rated as severe (irritability) and 4 patients (16%) discontinued the study due to TEAEs. During study phase 1, 2 patients discontinued due to TEAEs (mydriasis, vomiting); during study phase 2, 1 patient discontinued in the ATX/MPH group (supraventricular extrasystoles) and 1 patient in the ATX/PBO (irritability). The most common TEAEs were initial insomnia, vomiting, headache, nausea, and rhinitis. No deaths or serious adverse events occurred in the study.

**Table 1:** Frequency of treatment-emergent adverse events (TEAEs) from baseline to treatment endpoint by treatment group

**Event**	**ATX + MPH *N *= 9 *n *(%)**	**ATX + PBO *N *= 12 *n *(%)**
Initial insomnia	1 (11.1)	2 (16.7)
Rash	1 (11.1)	0 (0.0)
GI discomfort	1 (11.1)	0 (0.0)
Cardiac SE	1 (11.1)	0 (0.0)
Toothache	1 (11.1)	0 (0.0)
Vomiting	1 (11.1)	2 (16.7)
Abdominal pain	0 (0.0)	1 (8.3)
BP Increase	0 (0.0)	1 (8.3)
Hand fracture	0 (0.0)	1 (8.3)
Headache	0 (0.0)	2 (16.7)
Insomnia	0 (0.0)	1 (8.3)
Irritability	0 (0.0)	1 (8.3)
Nausea	0 (0.0)	2 (16.7)
Rhinitis	0 (0.0)	2 (16.7)

Key: GI, gastrointestinal; SE, supraventricular extrasystoles; BP, blood pressure

No significant differences were found between groups.

Categorical increases in vital signs occurred for 3 patients in the ATX/MPH group (1 systolic and diastolic blood pressures, 1 diastolic blood pressure, and 1 pulse rate) and for 2 patients in the ATX/PBO group (1 for pulse, 1 for systolic blood pressure) (Table 2). The sample sizes were too small to test for statistical significance. Overall, from study baseline to treatment endpoint, there was no statistically significant difference within patients or between groups in changes in blood pressure or pulse rate. Patients in the ATX/PBO group had a mean systolic BP change of -0.25 mm/Hg (sd = 10.0), a mean diastolic BP change of -1.83 (sd = 7.5), and a mean pulse change of -2.0 bpm (sd = 12.3). For patients in the ATX/MPH group, the mean change in systolic BP was 2.1 mm/Hg (sd = 11.2), in diastolic BP was 3.0 (sd = 8.5), and in pulse (sd = 5.0, sd = 12.6). There were significant differences between treatment groups for weight with patients who were augmented with placebo having a mean increase in weight of 0.84 kg compared with patients in the ATX/MPH group who had a mean decrease in weight of 0.89 kg (*P *≤ .05). The mean atomoxetine dose at endpoint was 1.07 mg/kg (sd = 0.12) for the ATX/MPH group and 1.09 (sd = 0.12) for the ATX/PBO group; the mean methylphenidate dose was 1.02 mg/kg for the group randomized to methylphenidate combination.

**Table 2:** Values of Vital Signs for Patients who met Criteria for Categorical Change

**Parameter**	**Augmentation Treatment**	**Augmentation Baseline**	**Categorical Change Value**	**Categorical Change**	**Duration of Augmentation**
Blood pressure (mm/Hg)					
Patient 1	MPH	84/56	110/86	D	1 week
Patient 2	MPH	110/53	124/74	S & D	6 weeks
Patient 3	PBO	108/78	110/90	D	2 weeks
					
Pulse Rate (bpm)					
Patient 1	MPH	84	114	P	2 weeks
Patient 2	PBO	78	112	P	2 weeks

Categorical change defined as follows: (1) for diastolic and systolic blood pressure, an increase of at least 5 mmHg to above the 95^th ^percentile based on age, gender, and height-adjusted National Institute of Health norms [15]; (2) for pulse, an increase of at least 25 to a value of at least 110. Abbreviations: MPH, methylphenidate; PBO placebo, D, met categorical change definition for diastolic blood pressure; S met categorical change definition for systolic blood pressure; P met categorical change definition for pulse.

### Efficacy outcomes

**Figure 1:**
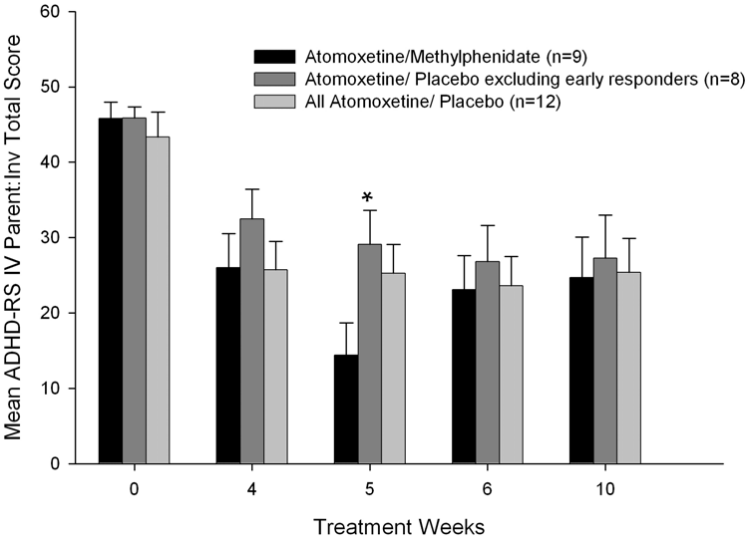
ADHDRS-IV-Parent:Inv mean total scores (SE) across treatment weeks. *P ≤ .05, atomoxetine and placebo (excluding early responders) vs atomoxetine and methylphenidate.

An overall treatment response to atomoxetine was found at the end of study phase 1 and study phase 2 (Figure 1), as demonstrated by the primary efficacy measure, the ADHDRS-IV-Parent: Inv total score. After 1 week of combination therapy, patients in the ATX/MPH group were significantly more improved than patients in the ATX/PBO group, excluding the early responders (*P *≤ .05), but there were no statistically significant group differences five weeks later at the end of the 10-week study. Atomoxetine treatment either alone or in combination with methylphenidate was associated with large effect sizes on the ADHDRS-IV-Parent: Inv total score (Table 3), which showed that atomoxetine treatment resulted in a mean improvement of 1.3 standard deviations above the baseline total score. Patients in the ATX/PBO group demonstrated significant overall improvement on the WREMB-PM subscale scores, but not the WREMB-AM subscale scores. Overall improvement associated with atomoxetine treatment was shown by changes in the CGI ratings and CPRS ratings within groups, but there was no significant incremental improvements within groups on these measures during the double-blind phase nor were there significant differences between groups at study endpoint (Table 3).

**Table 3:** Effect sizes for Efficacy Outcome Measures Across Treatment Phases

**Measures**	**ATX +MPH (*n *= 9)**	**ATX + PBO (excl. ER) (*n *= 8)**	**ALL ATX + PBO (*n *= 12)**
ADHD RS-IV-Parent:Inv T score			
Overall effect	1.3***	1.2**	1.3***
Incremental effect	0.05	0.18	0.03
WREMB-AM			
Overall effect	0.60	0.82	0.79
Incremental effect	0.43	0.07	0.11
WREMB-PM			
Overall effect	0.56	0.70	0.94*
Incremental effect	0.33	0.01	0.02
CGI-Severity			
Overall effect	1.3*	1.3**	1.4**
Incremental effect	.34	.38	0.25
Conner Parent Rating Scale			
Overall effect	1.2*	0.80**	0.98***
Incremental	0.18	0.09	0.07

Key: ER, Early responders; ADHD-RS-IV-Parent:Inv, ADHD Rating Scale Parent Report Investigator Rated; WREMB, Weekly Ratings Evening and Morning Behaviors. Note: Overall effect size was from baseline to study end-point (10 weeks ATX treatment); Incremental effect size from double-blind randomization to study end-point (6 weeks augmentation treatment).

*P ≤ .05, within group; **P ≤ .01, within group; *** *P *≤ .001, within group from baseline

After converting the ADHDRS-IV-Parent:Inv total score to a *T*-score, patients were classified by their response status as to whether they normalized or not. Across all patients, 38.1% did not meet normalization criteria during the study, 19.0% transiently normalized (met criteria at end of study phase 1), and 23.8% did not score in the normal range after study phase 1 but did at study endpoint. Overall, 42.9% either normalized by the end of study phase 1 and maintained improvement or had obtained normalization by the end of study phase 2.

## Discussion

To our knowledge, the present pilot study is the first placebo-controlled study of augmentation of atomoxetine with extended-release (OROS) methylphenidate. Although the study was initially intended to enroll a larger sample size, the inclusion eligibility criteria resulted in difficult recruitment. Specifically, many children who have experienced poor response to stimulants have already been treated with atomoxetine, and children who were naïve to treatment obviously could not be assessed for a prior history of stimulant response. The enrollment of the 25 children occurred across 5 centers; therefore, adding more research sites was not considered to be a viable solution, which then led to the study being closed. Given the small study sample, particularly in the combination treatment groups, the findings must be considered preliminary.

Analysis of the safety results did not reveal safety concerns that would preclude combining atomoxetine with extended-release methylphenidate over a 10-week period. There were no unexpected safety findings, and the observed changes in blood pressure were considered not to be clinically significant. Tolerability was favorable for both atomoxetine alone and the combination of atomoxetine and methylphenidate, as shown by the low frequency of treatment-emergent AEs as well as the overall 16% rate of discontinuation due to AEs.

The safety and tolerability findings from this study mirror previous findings of cardiovascular effects in patients who have received combined treatment as a portion of a clinical trial design. In a study optimizing the treatment of ADHD in 25 adults, the combination of atomoxetine and methylphenidate resulted in small statistically significant mean increases in pulse and heart rate. Categorical data analyses found one case of mild cardiac flutter in the context of normal pulse and blood pressure readings, and one case of systolic blood pressure increase of at least 15 mmHg to over 150 mmHg on one occasion (data on file, Eli Lilly and Company). In a study of healthy adults, the addition of atomoxetine to methylphenidate did not result in additive increases in heart rate or blood pressure [16]. For children with ADHD who were undergoing a switch from atomoxetine to methylphenidate, during the brief period of combined treatment, categorical changes in blood pressure and heart rate were within generally expected ranges [17].

With regard to efficacy, in a previous cross-over study comparing stimulants and atomoxetine, 45% of the children who did not respond to stimulants showed a ≥ 40% reduction in their ADHDRS-IV-Parent: Inv total score following atomoxetine treatment [18]. Consistent with this finding, in this sample of patients who had previously experienced an insufficient response to stimulant treatment, 43% demonstrated normalization of their ADHD symptoms following the 10-week atomoxetine treatment. On each measure, patients significantly improved in their core ADHD symptoms while taking atomoxetine and placebo during the first 4 weeks. The addition of methylphenidate initially significantly improved the patients' response in the first week, but this significant finding was evident only when excluding the early responders who were maintained on placebo. One of the early responders had significant variability by showing an initial response (32 point improvement) but then substantially worsening (22 points) by the end of treatment so that the patient was an eventual nonresponder. Overall, there was no significant increase in efficacy from the combined phase.

Given the small sample of this study, definitive conclusions on the effects of combining these medications would be premature, and the present findings can be applied only to children with an inadequate stimulant response. Nonetheless, the findings of the present study can provide guidance to the physician who is faced with patients with ADHD who are not responding sufficiently to stimulant treatment. Cardiovascular responses to the combination of atomoxetine and OROS methylphenidate were only minimally different from response to atomoxetine alone. These preliminary data suggest that atomoxetine appears to be safe when combined with extended-release (OROS) methylphenidate over the short-term (6 weeks). The addition of atomoxetine to enhance a partial response to stimulants maybe worth examining further in the future.

## Competing interests

Research was funded by Eli Lilly and Company, Indianapolis, IN. Dr. Carlson has received research support or has consulted with the following companies: Abbott Laboratories, Cephalon, Eli Lilly and Company, Janssen, McNeil, Otsuka, and Shire Pharmaceuticals. Dr. Dunn has received research support or has served on the speaker's bureaus of the following companies: AstraZeneca, Eli Lilly and Company, NIH, Otsuka, and Pfizer Pharmaceuticals. Drs. Kelsey, Ruff, Ball, and Allen and Ms. Ahrbecker are employees of and/or shareholders of Eli Lilly and Company.

## Authors' contributions

GAC, DD, AJA, DR, and DK and LA developed and implemented the clinical trial. GAC, DD, DK, SB, and AJA and LA developed the content outline, developed the design of analyses, and participated in interpretation of data. Statistical analyses were directed by DR. All authors participated in writing team meetings and contributed to the first draft. All authors have also read and approved the final manuscript version.

## References

[B1] Biederman J, Spencer T, Wilens T (2004). Evidence-based pharmacotherapy for attention-deficit hyperactivity disorder. Int J Neuropsychopharmacol.

[B2] Faraone SV, Biederman J (1998). Neurobiology of Attention-Deficit Hyperactivity Disorder. Biol Psychiatry.

[B3] Bhatara V, Feil M, Hoagwood K, Vitiello B, Zima B (2004). National trends in concomitant psychotropic medication with stimulants in pediatric visits: Practice versus knowledge. J Attent Disord.

[B4] Safer DJ, Zito JM, dosReis S (2003). Concomitant psychotropic medications for youths. Am J Psychiatry.

[B5] Carlson GA, Rapport MD, Kelly K, Grayson P, Pataki CS (1995). Methylphenidate and desipramine in hospitalized children with comorbid behavior and mood disorders: separate and combined effects on behavior and mood. J Child Adolesc Psychopharmacol.

[B6] Pataki CS, Carlson GA, Kelly KL, Rapport MD, Biancaniello TM (1993). Side effects of methylphenidate and desipramine alone and in combination in children. J Am Acad Child Adolesc Psychiatry.

[B7] Brown TE (2004). Atomoxetine and stimulants in combination for treatment of attention deficit hyperactivity disorder: Four case reports. J Child Adolesc Psychopharmacol.

[B8] Spencer T, Biederman J, Wilens T, Harding M, O'Donnell D, Griffin S (1996). Pharmacotherapy of attention-deficit hyperactivity disorder across the life cycle: A literature review. J Am Acad Child Adolesc Psychiatry.

[B9] DuPaul GJ, Power TJ, Anastopoulos AD, Reid R (1998). ADHD Rating Scale-IV: Checklists, Norms, and Clinical Impressions.

[B10] WMA. Declaration of Helsinki (2000). Ethical principles for medical research involving human subjects. World Medical Association. First adopted in 1964; most recent revision in 2000.

[B11] Kaufman J, Birmaher B, Brent D, Rao U, Flynn C, Moreci P, Williamson D, Ryan N (1997). Schedule for Affective Disorders and Schizophrenia for School-Age Children-Present and Lifetime Version (K-SADS-PL): initial reliability and validity data. J Am Acad Child Adolesc Psychiatry.

[B12] Guy W (1976). ECDEU assessment manual for psychopharmacology, revised.

[B13] Conners CK, Barkley RA (1985). Rating scales and checklists for child psychopharmacology. Psychopharmacol Bull.

[B14] Kelsey DK, Sumner CR, Casat CD, Coury DL, Quintana H, Saylor KE, Sutton VK, Gonzales J, Malcolm SK, Schuh KJ, Allen AJ (2004). Once daily atomoxetine treatment for children with attention deficit hyperactivity disorder, including an assessment of evening and morning behavior: A double-blind, placebo controlled trial. Pediatrics.

[B15] National High Blood Pressure Education Program Working Group (1996). Update on the 1987 task force report on high blood pressure in children and adolescents: A working group report from the national high blood pressure education program. Pediatrics.

[B16] Kelly RP, Yeo KP, Teng CH, Smith BP, Lowe S, Soon D, Read HA, Wise SD (2005). Hemodynamic effects of acute administration of atomoxetine and methylphenidate. J Clin Pharmacol.

[B17] Quintana H, Cherlin E, Duesenberg D, Bangs M, Ramsey J, Feldman P, Allen AJ, Kelsey D (2007). Transitioning from methylphenidate or amphetamine to atomoxetine in children and adolescents with attention-deficit/hyperactivity disorder – a preliminary tolerability and efficacy study. Clin Ther.

[B18] Newcorn J (2004). Atomoxetine and psychostimulants for ADHD: Is there differential response?.

